# Assessing the value of oligoclonal band count in multiple sclerosis: insights from a large cohort analysis

**DOI:** 10.3389/fneur.2026.1719470

**Published:** 2026-04-15

**Authors:** Konstantin F. Jendretzky, Ulrich Wurster, Franz Felix Konen, Katja Döring, Jule Finia Friedrich, Sandra Nay, Nora Möhn, Lea Grote-Levi, Martin W. Hümmert, Kurt-Wolfram Sühs, Philipp Schwenkenbecher, Stefan Gingele, Thomas Skripuletz

**Affiliations:** 1 Department of Neurology, Hannover Medical School, Hannover, Germany; 2 Institute for Diagnostic and Interventional Neuroradiology, Hannover Medical School, Hannover, Germany

**Keywords:** clinical manifestation, CSF, multiple sclerosis, oligoclonal bands, prognosis

## Abstract

Cerebrospinal fluid (CSF) specific oligoclonal bands (OCB) are a key diagnostic marker in multiple sclerosis (MS). Recent studies suggest that OCB reduction may reflect treatment efficacy and propose it as a potential endpoint in trials with chimeric antigen receptor (CAR) T-cell therapy. However, the prognostic value of baseline OCB count remains unclear. This study analyzed data from 454 persons (314 had MS, 140 clinically isolated syndrome). OCB analysis was performed by isoelectric focusing in polyacrylamide gel, followed by silver staining. The correlation of the number of OCBs with clinical and paraclinical parameters as well as follow-up outcomes was evaluated. Our results showed that in OCB positive persons with MS, the median number of CSF specific bands was 19. No significant correlation was found between OCB count and age, sex, initial EDSS, or MRI lesion load. OCB count also did not predict relapse risk or EDSS progression. However, strong correlations were observed with intrathecal synthesis of IgG and kappa free light chains. Therefore, our conclusion is that OCB count does not reflect clinical disease activity at diagnosis nor predict clinical progression within the observed median follow-up period of 9 months. Whether a decrease in OCB count indicates reduced humoral immune response in the CNS under certain therapies remains unclear and requires further prospective studies.

## 1 Background

Multiple sclerosis (MS) is an autoimmune-mediated disease of the central nervous system (CNS) with the migration of autoreactive B cells playing a key role in its pathophysiology (1). This triggers an immune cascade, leading to myelin damage and a heterogeneous clinical presentation (1). Due to the resulting diagnostic challenges, a need for standardized diagnostic criteria arose. The current standard criteria are the McDonald criteria which were first published in 2001 and were revised in 2005, 2010, 2017, and were again just recently updated in form of the 2024 McDonald criteria (1–6). These criteria often enable MS diagnosis at the first clinical event, in the case of only partial fulfillment, clinically isolated syndrome (CIS) is diagnosed (5). In 2017, the inclusion of cerebrospinal fluid (CSF)-restricted oligoclonal bands (OCB) as evidence for dissemination in time increased diagnostic sensitivity at the first demyelinating event, as confirmed in multiple real-world cohorts (5, 7–10). The 2024 criteria now further refined the diagnostic approach by, among other updates, accepting the kappa free light-chain (KFLC) index as an alternative to OCB for CSF evidence of dissemination in time. Pathophysiologically, OCB originate from CNS-migrated B cells, which undergo clonal expansion and differentiate into plasma cells, producing clonally restricted IgG (11, 12). These antibodies are detected as characteristic bands through isoelectric focusing and subsequent staining (11, 13). In addition to OCB, KFLC have emerged as a promising further biomarker (14, 15).

Recent MS studies explore the reduction in OCB count as a marker of CNS-targeted treatment efficacy. In this growing body of studies, usually an effect of a specific therapy on the reduction of the absolute number of CSF-specific OCB is described, which then typically sparks the discussion about a potentially more specific efficacy of the respective drug beyond the blood–brain barrier on the CNS of persons with MS (pwMS) (16–20). Particularly, therapeutic trials involving chimeric antigen receptor T (CAR T) cells, which are assumed to cross the blood brain barrier and target CNS B cells, use the reduction of the individual OCB count as a potential endpoint in therapeutic trials, again, to indirectly demonstrate B cell depletion (21). However, when focusing on the number of intrathecal OCB, only limited data exist on the prognostic value (22–24). Importantly, a distinction must be made between the presence or absence of CSF-restricted OCBs and the absolute number of OCBs when positive. While the presence of OCBs has been consistently associated with a higher risk of conversion from CIS to MS in large longitudinal cohorts (25, 26), the absolute count of OCB has been addressed by only few research groups, yielding ambivalent and partially contradictory results (22–24). The present study therefore aims to provide a comprehensive analysis of the number of CSF specific OCBs in relation to clinical and paraclinical parameters at the time of diagnosis and their prognostic value in pwMS. Specifically, we hypothesized that the baseline number of CSF-specific OCBs at diagnosis does not reflect clinical or MRI disease activity and does not predict short-term disability progression or relapse risk. The primary aim of this study was therefore to evaluate possible associations between baseline OCB count and clinical parameters [e.g., age, sex, Expanded Disability Status Scale (EDSS)], magnetic resonance imaging (MRI) lesion load, and follow-up outcomes. The secondary aim was to examine correlations between OCB count and quantitative measures of intrathecal humoral immune activity, including intrathecal IgG synthesis and the KFLC index.

## 2 Patients and methods

### 2.1 Patients

All individuals were evaluated at the Department of Neurology, Hannover Medical School (MHH, Hannover, Germany) between 2010 and 2017. Electronic medical records and laboratory databases were screened for individuals presenting with a first demyelinating event suggestive of chronic inflammatory CNS disorder. The 2017 McDonald criteria were applied retrospectively to confirm the diagnosis of MS or CIS. Inclusion criteria were age ≥18 years and CSF and serum analyses performed at the Neurochemistry Laboratory of MHH at the time of first presentation. Exclusion criteria comprised alternative diagnoses such as neuromyelitis optica spectrum disorder, MOG antibody-associated disease, infectious or neoplastic CNS disease, and cases lacking CSF analyses at MHH. All data were obtained retrospectively as part of routine diagnostic work-up. Follow-up data were obtained retrospectively from routine clinical visits. Because lumbar punctures and follow-up examinations were performed as clinically indicated, follow-up duration varied considerably between individuals, resulting in a median follow-up of 9 months. The first demyelinating symptom was defined according to the initial neurological presentation documented by the treating neurologist. Disability was quantified using the EDSS as assessed at the time of lumbar puncture and at follow-up visits based on standardized neurological examinations. Follow-up data were retrieved from subsequent outpatient or inpatient visits documented in the electronic hospital system. This study partially included persons previously described in earlier studies (27, 28).

### 2.2 CSF and serum analytical procedures

Paired CSF and serum samples were analyzed at the Neurochemistry Laboratory of the Department of Neurology at Hannover Medical School as part of the routine diagnostics. CSF cell count was evaluated manually using a Fuchs-Rosenthal chamber, with a normal CSF cell count defined as <5 cells/μL. Total CSF protein was measured via the Bradford method (threshold: 500 mg/L). Immunoglobulin (IgG, IgA, IgM) and albumin levels in CSF and serum were assessed via latex-enhanced kinetic nephelometry (Beckman Coulter IMMAGE). The CSF/serum albumin ratio was calculated using the age-adjusted formula *QAlb = 4 + (age in years/15)*. To detect intrathecal synthesis, IgG, IgA, and IgM ratios were plotted against albumin ratios according to Reiber (29).

**Figure 1:**
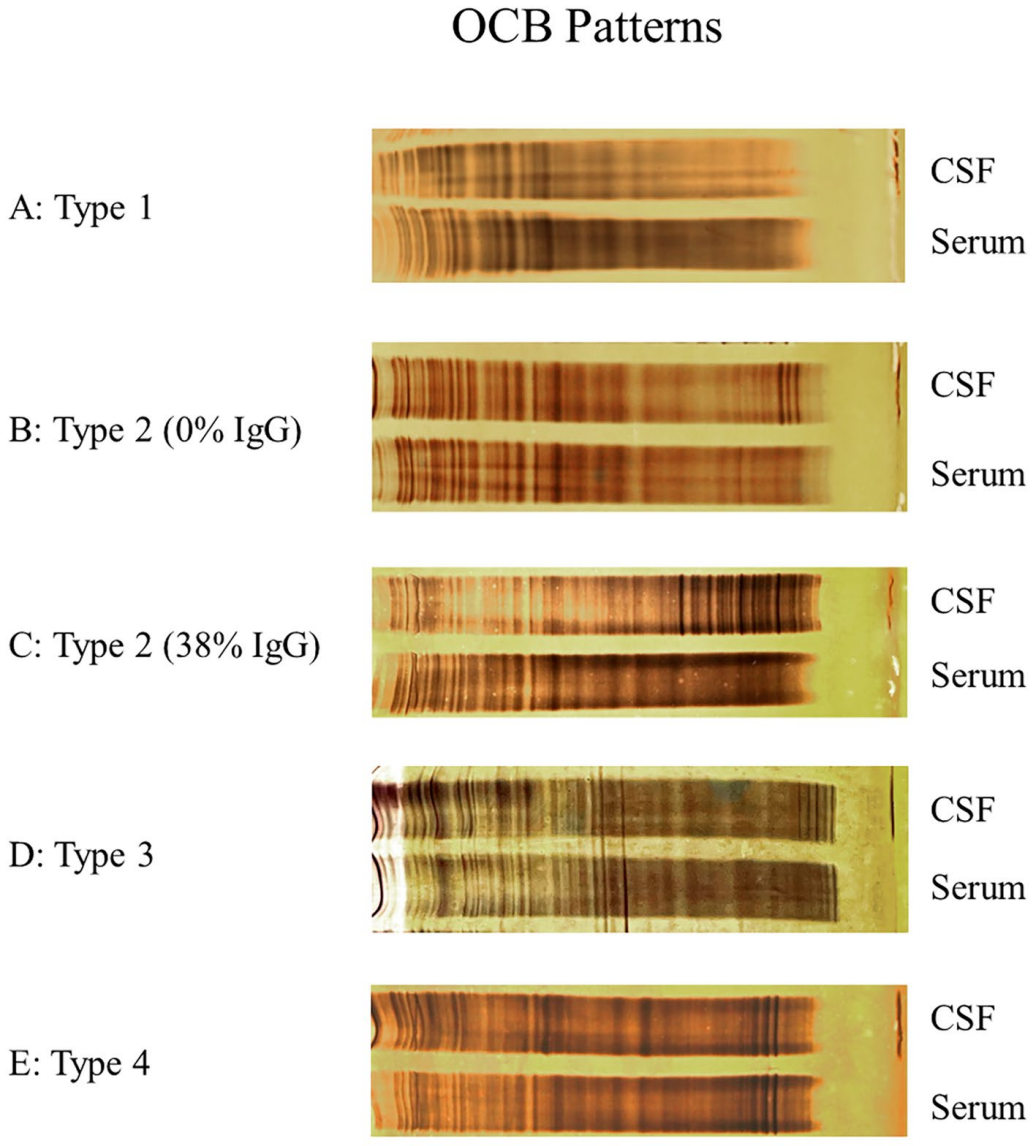
Picture of oligoclonal bands (OCB) patterns prepared with silver staining after isoelectric focusing on polyacrylamide gel of serum and cerebrospinal fluid (CSF), showing OCB type 1 (A), type 2 of a person with no detectable intrathecal immunoglobulin G (IgG) synthesis in CSF according to IgG Reiber diagram (B), type 2 of a person with detectable intrathecal immunoglobulin G (IgG) synthesis in CSF in the Reiber diagram (C), type 3 (D), and type 4 (E).

OCB were detected by performing isoelectric focusing on polyacrylamide gels, followed by silver staining. Equal amounts of IgG (standardized to 20 mg/L) from CSF and serum samples were loaded onto the gel. The isoelectric focusing separates proteins based on their isoelectric points to differentiate OCB present in the CSF and absent in the serum. After isoelectric focusing, silver staining was used to visualize the OCB patterns (Figure 1). Patterns were classified by an author’s experienced rater (UW) blinded to patient data, following European consensus guidelines (13). To determine the number of OCB exclusively detectable in the CSF, the bands in both the serum and CSF were counted separately. The net value was calculated by identifying the number of bands only visible within CSF. The presence of ≥2 oligoclonal bands solely in the CSF was considered a positive finding.

To enable a comparison between the number of CSF-specific oligoclonal bands and the amount of IgG produced in the CNS, the actual quantity of intrathecally produced IgG was calculated. For this purpose, the formula (Reiber IgG synthesis/100)* total measured CSF IgG was applied.

Serum and CSF concentrations of KFLC were measured using nephelometry with the N Latex FLC kappa kit (Siemens Healthcare Diagnostics Products GmbH), following the manufacturer’s instructions on an Atellica Neph 630 System (Siemens Healthcare Diagnostics Products GmbH). KFLC index was calculated using the formula: *(KFLC_CSF_/KFLC_serum_)/(albumin_CSF_/albumin_serum_)*. Values above the threshold of 6.1 were considered elevated.

### 2.3 Magnetic resonance imaging

All persons underwent brain MRI, including at least T1-weighted, T2-weighted, and gadolinium (Gd)-enhanced T1-weighted sequences, performed on MR systems with a magnetic field strength of at least 1.5 T. For a subset of 52 individuals, the exact number of T2 hyperintense lesions, as well as the number of Gd-enhanced T1 lesions and spinal cord lesions, if applicable, was recorded.

### 2.4 Statistics

Data analysis was performed using GraphPad Prism 8.43 (GraphPad Software, United States) and SPSS (version 26, Armonk, NY, United States). The D’Agostino & Pearson omnibus normality test was applied to assess Gaussian distribution. For comparisons among three or more groups with non-parametric distributions, the Kruskal–Wallis test was employed, followed by Dunn’s *post-hoc* test. The Mann–Whitney *U* test was used to compare two groups with non-parametric data samples. Receiver operating characteristic (ROC) curves were generated to evaluate the diagnostic performance of OCB count, KFLC index, and intrathecal IgG synthesis in differentiating MS from CIS. To assess the combined diagnostic value of OCB and KFLC index, a multivariable logistic regression model including both markers was fitted, and predicted probabilities were used to generate ROC curves. Sensitivity, specificity, and area under the curve (AUC) were calculated in a subset of 269 individuals with complete data for all three markers. Spearman’s correlation assessed relationships between ordinal or non-normally distributed variables. Fisher’s exact test examined categorical variable independence. To evaluate the influence of age, sex, and OCB count on relapse occurrences during the follow-up period, Cox regression analyses were performed, with a median split applied to age. A binomial regression was conducted to identify factors potentially impacting EDSS worsening over the follow-up period. Non-normally distributed results were presented as median and interquartile range (IQR). Unstandardized regression coefficients (B) and hazard ratios (HR) were used with 95% confidence intervals (CI). Statistical significance was *p* < 0.05.

## 3 Results

### 3.1 Demographic and clinical characteristics

The study included 454 persons of which 67% were women, 314 diagnosed with MS (66% women) and 140 with CIS (69% women). The median age was 32.5 years (IQR 26–42) for pwMS and 36 years (IQR 28–45) for persons with a CIS diagnosis (pwCIS). At initial diagnosis, the median EDSS score was 2 (IQR 1.5–2.5) for pwMS and 2 (IQR 1.0–2.5) for pwCIS. Follow-up data were available for 276 persons, with a median duration of 9 months (IQR 2–27) for pwMS and 8 months (IQR 2–20) for pwCIS. No significant differences were found between the cohorts in terms of age, sex distribution, initial EDSS scores, or follow-up duration (Table 1).

**Table 1:** Characteristics of included persons.

Parameter	MS (*n* = 314)	CIS (*n* = 140)
Female (%)	207/314 (66%)	97/140 (69%)
Age—median (IQR)	32.5 (26–42)	36.0 (28–45)
EDSS—median (IQR)	2.0 (1.5–2.5)	2.0 (1.0–2.5)
Follow-up available, *n* (%)	210/314 (67%)	66/140 (47%)
Follow-up duration—median (IQR)	9 (2–27)	8 (2–20)

MS, multiple sclerosis; CIS, clinically isolated syndrome; IQR, 25–75 interquartile range, EDSS, Expanded Disability Status Scale.

### 3.2 Diagnostic performance and correlation of biomarkers

**Figure 2:**
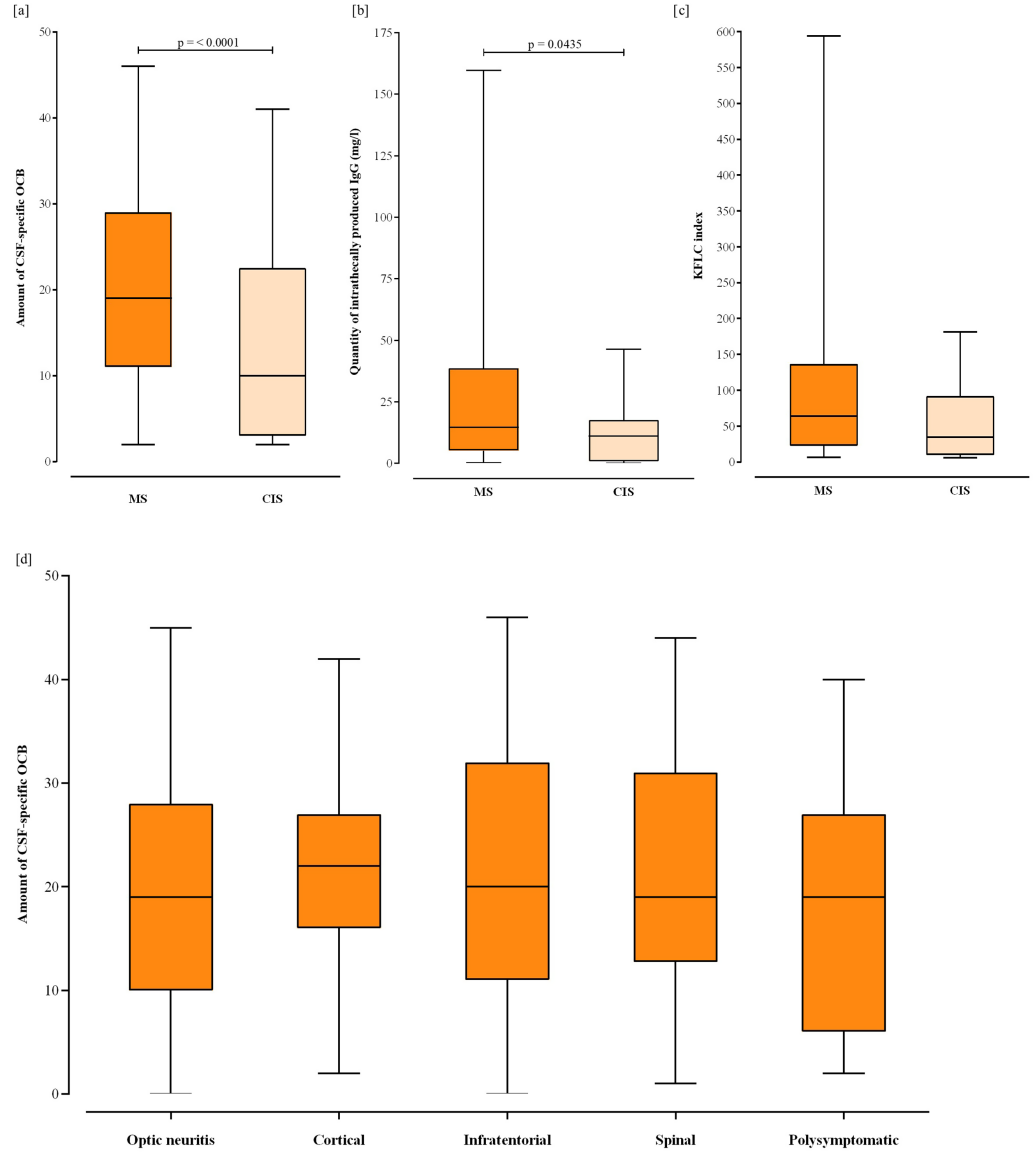
Box plots illustrating **(a)** the number of CSF-specific oligoclonal bands (OCB) of OCB positive persons with MS and CIS, **(b)** intrathecal produced amount of IgG of persons with MS and CIS with detectable IgG-synthesis, according to Reiber’s diagram, and **(c)** KFLC index of all persons with MS and CIS with KFLC index >6.1. Panel **(d)** shows OCB count cross across different clinical presentation types. Boxes represent interquartile range with horizontal line within the box representing the median. Lower and upper whiskers represent min. and max. values. MS, multiple sclerosis; CIS, clinically isolated syndrome; OCB, oligoclonal bands; CSF, cerebrospinal fluid; IgG, immunoglobulin G; KFLC, kappa free light chains.

OCB positivity was observed in 311 of the total 314 pwMS (99% of the MS cohort) and in 41 of 140 pwCIS (29.3%) (Table 2). To test for differences of the total number of OCB only detectable in CSF, the OCB results of all persons were counted manually as described in the methods section. Among OCB-positive pwMS, the median number of net OCB was 19 (IQR 11–29) compared to 10 (IQR 3–22) in pwCIS (*p* = <0.0001) (Figure 2a).

**Table 2:** Presence of biomarker positivity in persons with MS and CIS.

Biomarker	MS (*n* = 314)	CIS (*n* = 140)
OCB positive, *n*/total (%)	311/314 (99%)	41/140 (29.3%)
Detection of intrathecal IgG synthesis according to Reiber, *n*/total (%)	195/314 (62.1%)	17/140 (12.1%)
KFLC index >6.1, *n*/total (%)	154/167 (92.2%)	24/102 (23.5%)

MS, multiple sclerosis; CIS, clinically isolated syndrome; OCB, oligoclonal bands; IgG, immunoglobin G; KFLC, kappa free light chain.

Intrathecal IgG synthesis as detected by Reiber IgG diagram was present in 195/314 (62%) pwMS and 17 of 140 pwCIS (12.1%) (Table 2). One hundred and sixteen of the one hundred and nineteen pwMS without detectable IgG synthesis according to Reiber (36.9% of all 314 pwMS) had a positive OCB result. In those with detectable IgG synthesis, the median level of intrathecally produced IgG was 14.7 mg/L (IQR 5.2–38.7) in pwMS and 11.1 mg/L (IQR 0.9–17.2) in pwCIS (*p* = 0.0435) (Figure 2b).

In a subset of 167 pwMS and 102 pwCIS, KFLC were obtained. Within this subgroup, an elevated KFLC index (>6.1) was noted in 154 out of 167 pwMS (92%) and in 24 pwCIS (23.5%) (Table 2). Of those with a KFLC index >6.1, the median KFLC index was 63.9 (IQR 23.3–135.4) in pwMS compared to 34.9 (IQR 10.2–87.2) in pwCIS. Here, there was no significant difference (Figure 2c).

**Figure 3:**
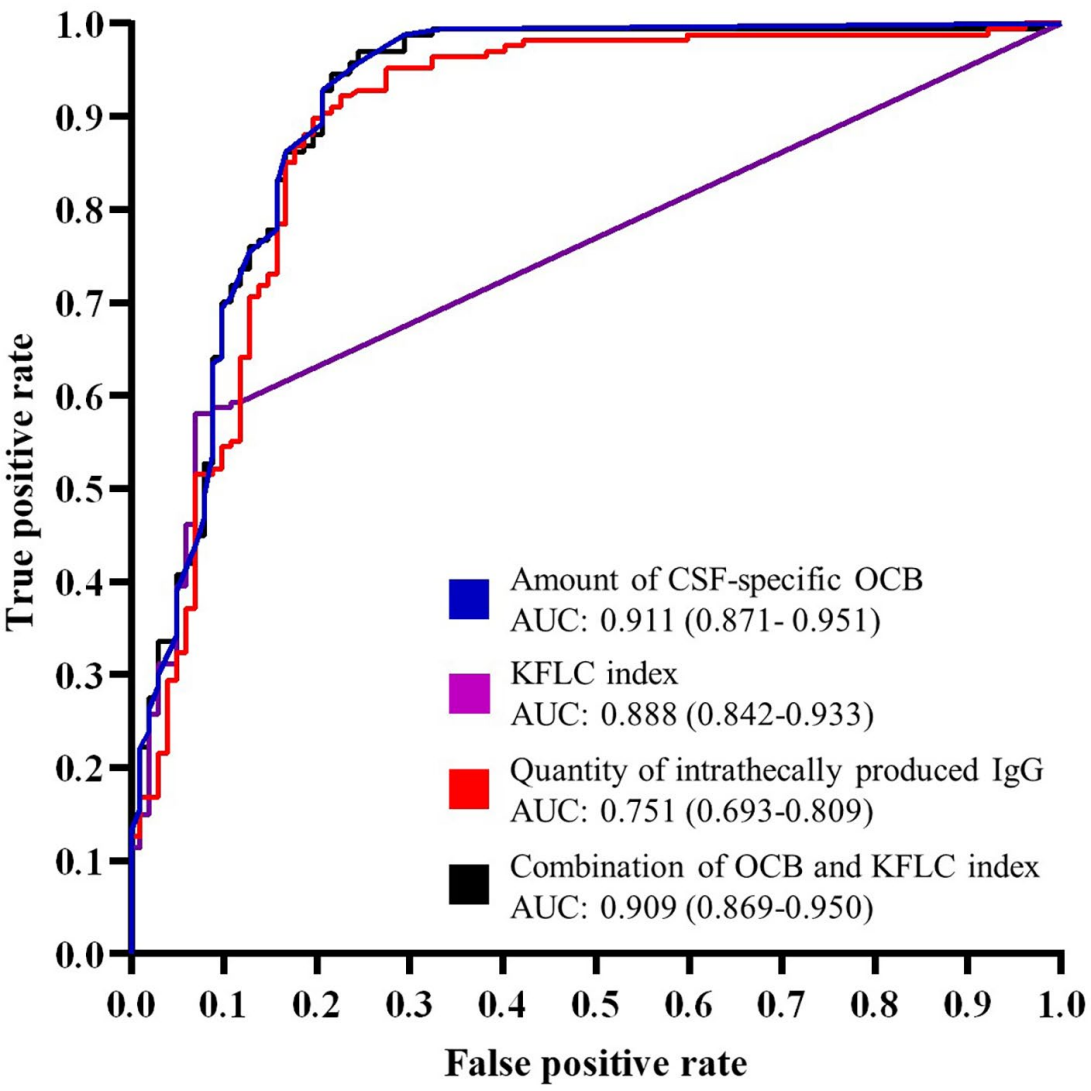
Receiver operating characteristic (ROC) curves for the differentiation of MS and CIS (*n* = 269). The plot illustrates the diagnostic accuracy of OCB count, KFLC index, intrathecal IgG synthesis according to Reiber, and the combination of OCB count and KFLC index. The area under the curve (AUC) with corresponding 95% confidence interval (CI) for each methodology is indicated in the legend. The distinct inflection point in the intrathecal IgG curve reflects the high proportion of individuals with a calculated IgG synthesis of 0.0 mg/L, where the methodology reaches its limit of continuous discrimination. MS, multiple sclerosis; CIS, clinically isolated syndrome; OCB, oligoclonal bands; KFLC, kappa free light chain; IgG, immunoglobulin G.

As shown in Table 3, 12 out of 13 pwMS who were negative for the KFLC index were correctly identified by positive IgG-OCBs. Only one pwMS was KFLC positive but OCB negative. To further compare the diagnostic value, ROC analyses were performed (Figure 3). IgG-OCB positivity showed the highest sensitivity (98.8%) and the largest AUC (0.911) in differentiating MS from CIS, followed by the KFLC index (92.2%, AUC 0.888) and quantitative IgG synthesis (59.3%, AUC 0.751) (Table 4). Notably, the combination of IgG-OCB and KFLC index increased the sensitivity to 99.4% (Table 4).

**Table 3:** Diagnostic concordance MS vs. CIS.

Disease category	Both positive	Only OCB+	Only KFLC+	Both negative	Total
MS	153 (91.6%)	12 (7.2%)	1 (0.6%)	1 (0.6%)	167
CIS	21 (20.6%)	9 (8.8%)	3 (2.9%)	69 (67.6%)	102

Data are presented for a subset of 269 individuals with available results for both parameters. MS, multiple sclerosis; CIS, clinically isolated syndrome; OCB, oligoclonal bands; KFLC, kappa free light chain.

**Table 4:** Diagnostic performance of the different biomarkers MS vs. CIS.

Method	Sensitivity (%)	Specificity (%)	AUC (95% CI)
OCB positivity	98.8	70.6	0.911 (0.871–0.951)
KFLC index (>6.1)	92.2	76.5	0.888 (0.842–0.933)
Intrathecal IgG (>0)	59.3	88.2	0.751 (0.693–0.809)
Combination of OCB and KFLC index	99.4	67.6	0.909 (0.869–0.950)

Sensitivity, specificity, and area under the curve (AUC) are shown for OCB positivity, KFLC index, intrathecal IgG synthesis, and the combination of OCB and KFLC index. Analyses were performed on a subset of 269 persons with complete datasets for all markers. MS, multiple sclerosis; CIS, clinically isolated syndrome; OCB, oligoclonal bands; KFLC, kappa free light chain; IgG, immunoglobulin G; CI, confidence interval.

**Figure 4:**
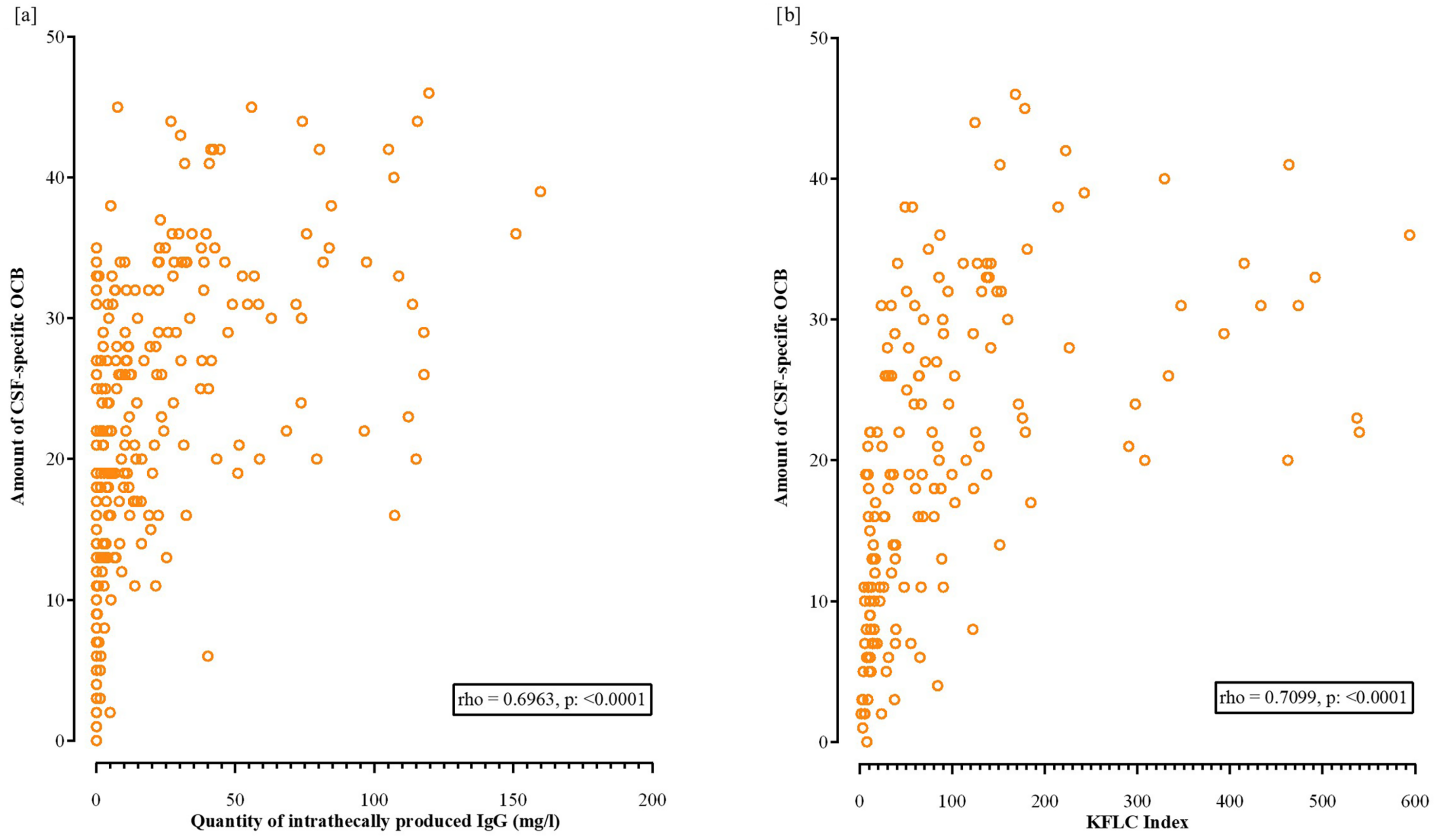
Scatter plots showing the positive correlations between the number of oligoclonal bands (OCB) in CSF with **(a)** intrathecal IgG levels (rho = 0.6963, *p* < 0.0001) and **(b)** KFLC index (rho = 0.7099, *p* < 0.0001). MS, multiple sclerosis; OCB, oligoclonal bands; CSF, cerebrospinal fluid; KFLC, kappa free light chains; EDSS, IgG, immunoglobulin G.

Spearman correlation analysis showed a positive correlation between the number of OCB and intrathecal IgG levels (*ρ* = 0.6963, *p* = <0.0001), and between the number of OCB and the KFLC index (*ρ* = 0.7099, *p* = <0.0001) (Figures 4a,b).

### 3.3 Correlation between number of OCB and clinical parameters

To identify possible correlations between number of OCB and clinical parameters we examined whether the number of OCB was associated with age or sex. Median OCB count was not significantly different between male and female persons with a median of 19 in female, (*n* = 207) and 20 for male persons (*n* = 107). A Spearman correlation analysis was used to test for a possible relationship between age and the number of OCB, yielding a correlation coefficient of −0.04624 (95% CI: −0.1593 to 0.06805, *p* = 0.4142), which suggested no significant association between age and OCB count. Spearman’s rank correlation coefficient between the number of OCB and the initial EDSS was −0.1095 (95% CI: −0.2477 to 0.03313, *p* = 0.121), again indicating no significant association between these variables in pwMS.

Additionally, we evaluated the relationship between OCB count and type of clinical presentation at the time of diagnosis, categorized into optic neuritis (*n* = 119), cortical (*n* = 43), infratentorial (*n* = 51), spinal (*n* = 74), and polysymptomatic forms (*n* = 27). No significant differences were found across these categories. Further pairwise comparisons also revealed no significant differences between any specific presentation types (Figure 2d).

### 3.4 Correlation of number of OCB and MRI parameters

For a subset of 52 pwMS the exact number of MRI lesions was obtained. Of these, 17 (32.7%) had Gd-positive lesions, and 15 (28.8%) had spinal lesions. Spearman correlations revealed no significant correlations between OCB count and any MRI parameter (cranial T2: rho = 0.1575, *p* = 0.2647; Gd-positive: rho = −0.1444, *p* = 0.312; spinal: rho = −0.08288, *p* = 0.6208). Mann–Whitney *U*-tests showed no significant differences in OCB count between pwMS with and without Gd-positive or spinal lesions (Gd+: median 17 vs. Gd−: 24, *p* = 0.2008; spinal+: 16 vs. spinal−: 22, *p* = 0.5006). Binary logistic regressions showed a non-significant trend between OCB count and presence of Gd lesions (*p* = 0.059, B = −0.0584), but no association with spinal lesions (*p* = 0.884). Age-stratified analyses also showed no significant results.

### 3.5 Correlation of number of OCB and follow-up parameters

To assess if the number of CSF-specific OCB correlated with follow-up parameters in pwMS, a Cox regression analysis was conducted. Primary focus was on the effect of OCB count on relapse risk, with additional parameters including age and sex. Results from univariable and multivariable analyses for the entire cohort, as well as age-adjusted subgroups, are summarized in Table 5. Within the first analysis only age emerged as significant follow-up parameter, thus, to adjust for age, a median split was performed. Within the stratified analyses, no significant results could be seen. Additionally, a binary logistic regression was conducted to assess the influence of OCB count on EDSS worsening. For this, a differential EDSS score was calculated for each person, defined as the difference between the EDSS at first presentation and the EDSS at last follow-up, with EDSS worsening classified as a difference of at least 0.5. Again, additional parameters included were age and sex. To adjust for possible influences of age an age-adjusted analysis was performed as well. The analysis showed no significant associations between age, sex, or OCB count with EDSS worsening (see Table 5).

**Table 5:** Correlation of number of OCB and follow-up parameters in persons with MS.

Model	Number of CSF OCB	Age (per year)	Sex (female vs. male)
Cox regression: relapse-free survival [results shown as HR (95% CI, *p*-value)]			
Univariable	1.00 (0.98–1.03, *p* = 0.677)	**0.96 (0.93–0.98, *p* < 0.001)**	0.98 (0.63–1.54, *p* = 0.943)
Multivariable	1.00 (0.98–1.02, *p* = 0.961)	**0.96 (0.93–0.98, *p* < 0.001)**	0.88 (0.55–1.40, *p* = 0.585)
Age-adjusted (persons >32) univariable	1.03 (0.99–1.06, *p* = 0.137)	0.97 (0.92–1.01, *p* = 0.166)	1.21 (0.56–2.63, *p* = 0.631)
Age-adjusted (persons >32) multivariable	1.02 (0.99–1.06, *p* = 0.215)	0.97 (0.93–1.02, *p* = 0.244)	1.26 (0.58–2.75, *p* = 0.559)
Age-adjusted (persons ≤32) univariable	0.98 (0.96–1.01, *p* = 0.248)	0.95 (0.89–1.02, *p* = 0.197)	0.76 (0.43–1.34, *p* = 0.341)
Age-adjusted (persons ≤32) multivariable	0.99 (0.96–1.02, *p* = 0.484)	0.95 (0.89–1.03, *p* = 0.207)	0.77 (0.42–1.41, *p* = 0.405)
Logistic regression: EDSS worsening [results shown as regression coefficients (B) (95% CI, *p*-value)]			
Whole cohort	0.017 (−0.04–0.08, *p* = 0.582)	−0.007 (−0.07–0.06, *p* = 0.845)	−0.120 (−1.13–0.89, *p* = 0.876)
Age-adjusted (persons ≤32)	−0.033 (−0.13–0.06, *p* = 0.500)	0.123 (−0.15–0.39, *p* = 0.377)	0.053 (−0.23–0.34, *p* = 0.958)
Age-adjusted (persons >32)	0.070 (−0.06–0.20, *p* = 0.238)	0.057 (−0.13–0.25, *p* = 0.531)	−0.448 (−2.88–1.98, *p* = 0.728)

EDSS worsening was defined as a difference of at least 0.5, calculated as the EDSS at first presentation subtracted from the EDSS at last follow-up. Cox regression analysis provided hazard ratios (HR) with 95% confidence intervals (CI) and *p*-values for univariable and multivariable models, including age-adjusted subgroups. Bold values indicate statistical significance (*p* < 0.05). Logistic regression results for EDSS worsening are shown as regression coefficients (B) with 95% CI and *p*-values. In the initial analysis, only age emerged as a significant follow-up parameter, prompting an age adjustment using a median split. The subsequent analyses revealed no significant associations. MS, multiple sclerosis; OCB, oligoclonal bands; EDSS, Expanded Disability Status Scale; HR, hazard ratio; CI, confidence interval.

### 3.6 Change of OCB count depending on therapy modality

To determine if OCB might disappear during treatment with highly effective therapeutic agents, an additional cohort of persons who underwent lumbar puncture while receiving CD20-depleting drugs or natalizumab was analyzed. In this cohort, OCB remained positive in 10 of 11 persons treated with natalizumab (median treatment duration: 25 months, IQR 8.5–50.75) and in 9 of 10 persons treated with anti-CD20 antibodies (median 13 months, IQR 6.25–26.25) (see Table 6).

**Table 6:** OCB positivity and intrathecal IgG synthesis in persons treated with natalizumab and CD-20-depleting agents.

Therapy	Total persons	Median treatment duration, months (IQR)	OCB type 2 or 3, *n* (%)	Presence of intrathecal IgG synthesis, *n* (%)
Natalizumab	11	25 (8.5–50.75)	10 (90.9%)	7 (63.6%)
CD-20-depleting therapy	10	13 (6.25–26.25)	9 (90.0%)	6 (60.0%)

Intrathecal IgG synthesis was calculated according to Reiber. OCB, oligoclonal bands; IgG, immunoglobin G.

**Figure 5:**
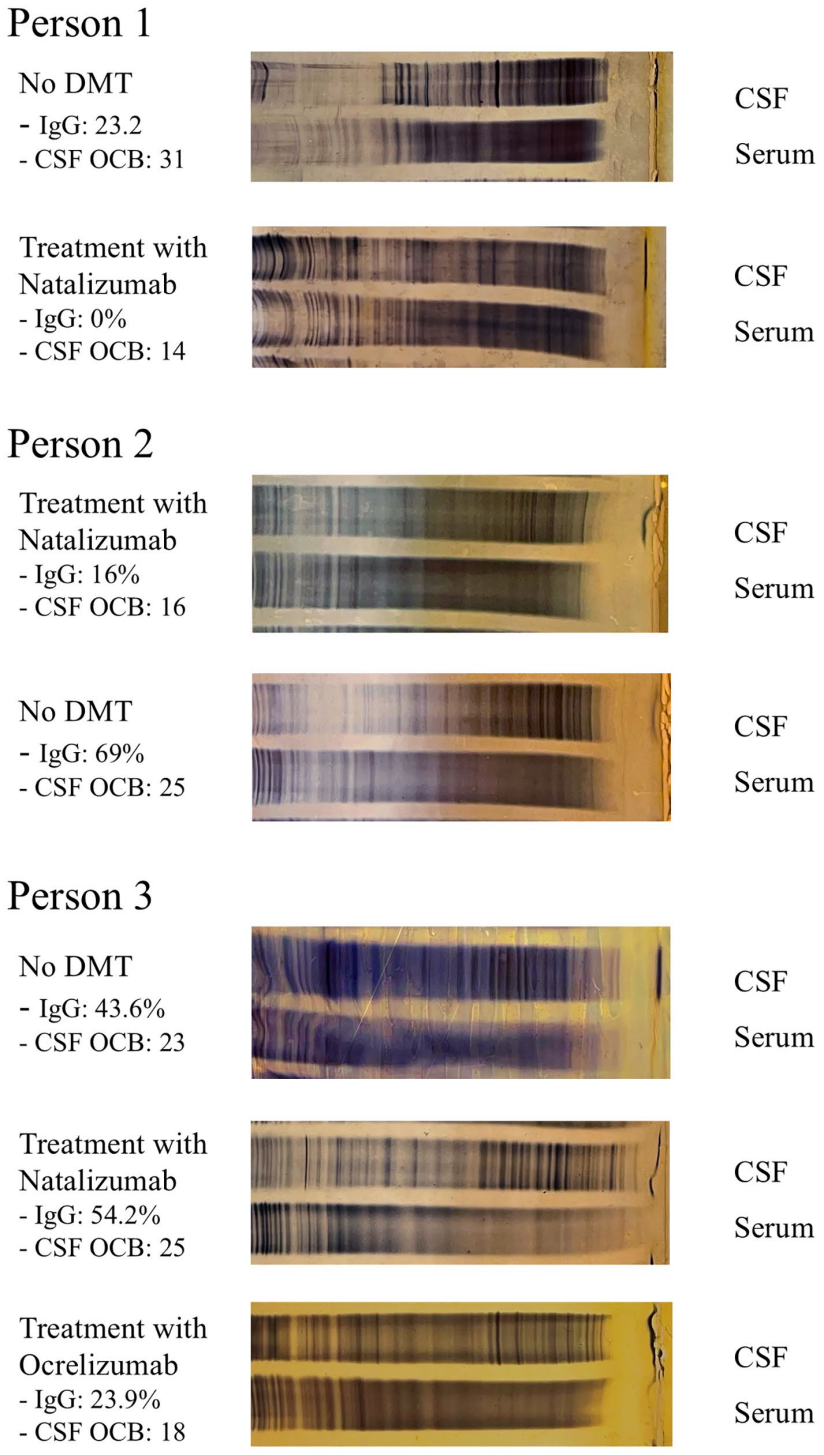
Changes in OCB count under different therapeutic agents. Individual OCB profiles and accompanying IgG synthesis according to Reiber are shown in three persons with MS with repeated CSF analyses under varying therapies. Person 1: OCB at initial disease manifestation (prior to DMT) and during natalizumab therapy. Person 2: OCB during and after discontinuation of natalizumab. Person 3: Before DMT, during natalizumab therapy, and after switching to ocrelizumab due to relapse activity. DMT, disease-modifying therapy; IgG, amount of intrathecal immunoglobulin G production according to Reiber.

In a subsequent analysis of three persons, specific changes in net OCB count were tracked under the different therapeutic modalities. Here, in person 1, number of CSF-specific OCB was assessed during first manifestation of the disease and thus before the initiation of a disease-modifying therapy (DMT) and again after 28 months of natalizumab therapy, showing a CSF-specific reduction in the number of OCB following natalizumab initiation. In person 2 who had received natalizumab for approximately 8 years, the amount of OCB was evaluated at the end of that treatment span and after discontinuation of natalizumab, revealing a subsequent increase in bands following the cessation of therapy. And in person 3, OCB count was assessed at first manifestation of MS, therefore before DMT use, secondly because of relapse activity during 11 months of natalizumab therapy, and 2 months after switching to ocrelizumab. During relapse under natalizumab treatment, a slight increase in CSF OCB number was observed compared to the initial CSF findings. After switching to ocrelizumab, a marked decrease in net OCB count was noted. Taken together, these findings indicate a mild to moderate reversible decrease in the number of CSF-specific OCB during treatment with different high-efficacy DMT without disappearance of OCB in these persons (Figure 5).

## 4 Discussion

The number of CSF-specific OCB has regained attention in research, partially due to their utilization as a parameter to evaluate the CNS effects of different therapies, particularly in clinical trials involving CAR T cells where they are used to indirectly demonstrate presumed depletion of B cells in the CNS (17, 19, 21). However, large-scale studies exploring the correlation of the count of CSF-specific OCB and markers of disease activity and treatment response in pwMS are limited. This scarcity may be attributed to the complexities involved in accurately counting bands and significant variations in the sensitivity of the different methods used for OCB detection (30–32). There are only few large studies that have investigated overall levels of CSF-specific OCB across different cohorts. Many of these studies are outdated, and others included pwMS undergoing various therapeutic regimens, which may limit comparability across the analyzed cohorts, as immunomodulatory treatments can affect the number of oligoclonal bands (22–24, 33). Here, we investigated a large cohort of pwMS at the time of their initial diagnosis, prior to any immunomodulatory treatment. Among pwMS with positive OCB, we observed a high median CSF specific OCB number of 19 bands, exceeding values reported in comparable studies (22, 23). Our findings identify a higher sensitivity of IgG-OCBs (99%) compared to the KFLC index (92%). In our laboratory, isoelectric focusing followed by high-sensitivity silver staining is the standard procedure. Unlike automated immunoblotting or Coomassie staining, this method, when performed by experienced personnel, allows for the detection of very low-level intrathecal IgG fractions (30–32). This highlights that OCBs remain a superiorly sensitive marker in specialized centers, supporting their continued role as a gold standard in the 2024 McDonald criteria (6). In further analyses, we demonstrated a strong correlation between the number of OCB and the amount of intrathecally produced IgG and KFLC. This was anticipated, given their shared origin and prior reports of such associations (14, 34). However, the fact that OCB were found in 36.9% of individuals without IgG detection by Reiber’s diagram and in 7.2% of pwMS with a negative KFLC index, was noteworthy, underlining both the sensitivity of our method and the qualitative diagnostic value of OCB.

Although the presence of OCB, intrathecal IgG synthesis, and KFLC are widely accepted as diagnostic markers, their prognostic utility remains debated (24–26, 33–37). Studies which investigated the role of the OCB count have also yielded partially inconclusive results (22–24, 33). In our study, there was consistently no significant correlation between the number of CSF-specific OCB and relevant clinical or paraclinical parameters. OCB were not significantly associated with initial disability as measured by the EDSS score, nor with the total number of T2 lesions on MRI. Similarly, follow-up parameters such as the occurrence of relapses and EDSS worsening showed no significant correlations with the number of OCB. This suggests that while OCB remain critically important for diagnosing MS, they do not appear to serve as reliable prognostic markers within the limited observation period of our study.

We investigated changes in OCB positivity in an additional cohort of 21 persons undergoing treatment with highly effective therapies, including natalizumab and anti-CD20 monoclonal antibodies. In 19 of 21 persons, OCB remained detectable and additionally, we noted an increase in OCB bands after discontinuing natalizumab, suggesting an individual specific OCB profile akin to an “MS fingerprint.” However, our study did not include a sufficiently large number of persons with OCB analyses both before and after high-efficacy therapy. Therefore, we cannot conclusively determine whether this marker might be useful for monitoring disease activity. Finally, and probably most noteworthy is that an evaluation of the OCB has now been described for several therapies, whereby at least we were able to show that the clinical significance from the standpoint of the first demyelinating event remains low. This implicates that it should be questioned whether and how a reduction in OCB under different therapies should best be interpreted. Thus, prospective and long-term studies with structured follow-up would be necessary to further validate these findings and better understand the potential clinical implications of the CNS specific OCB count.

In conclusion, this extensive and methodologically robust study demonstrates that, while CSF specific OCB are a highly sensitive marker for the presence of intrathecal IgG production, again underscoring the relevance as a diagnostic parameter, the amount of CSF-specific OCB at the time of initial diagnosis does not serve as a reliable marker for disease activity or prognosis. The number of OCB did not correlate with clinical or paraclinical parameters, nor did it correlate with subsequent disease progression, including relapses and disability development. Furthermore, our data demonstrate that the combination of OCB and KFLC index maximizes diagnostic safety, reaching a sensitivity of 99.4%. The incremental yield of OCBs in KFLC-negative pwMS (7.2% of the subset) suggests that both markers should ideally be used in a complementary manner to avoid missing cases with low-level humoral activity.

### 4.1 Limitations

Despite the comprehensive analysis of this large cohort, some limitations must be considered. First, this study is of a retrospective character. Furthermore, the number of individuals with detailed MRI parameters was relatively small (*n* = 52), which limits the statistical power to detect potentially subtle associations between OCB count and specific MRI outcomes such as lesion load or spinal involvement. And lastly, the follow-up period was relatively short (median 9 months), as lumbar punctures and follow-up visits were performed based on clinical indication rather than predefined study intervals. Consequently, long-term prognostic interpretations should be made with caution.

## Data Availability

The raw data supporting the conclusions of this article will be made available by the authors, without undue reservation.
